# Effects of two formulations containing *Phyllanthus emblica* and *Tinospora cordifolia* with and without *Ocimum sanctum* in immunocompromised mice

**DOI:** 10.1016/j.jaim.2021.06.021

**Published:** 2021-11-17

**Authors:** Harshad Malve, Dipti More, Ashwini More

**Affiliations:** a Department of Pharmacology, Vedanta Institute of Medical Sciences, Dahanu, India; b Department of Pediatrics, Lokmanya Tilak Municipal General Hospital and Medical College, Sion, Mumbai, India; c Department of Medicine, Vedanta Institute of Medical Sciences, Dahanu, India

**Keywords:** *Bhavana*, Hemisplenectomy, Immunomodulator, *Ocimum sanctum*, *Phyllanthus emblica*, *Rasayana*, *Tinospora cordifolia*

## Abstract

**Background:**

Current pandemic has led us to explore the role of traditional system of medicine to look for formulations that enhance immunity.

**Objective:**

The obejctive of this experimental study was to evaluate the immunomodulatory effects of two formulations, *Tinospora cordifolia* (Tc) and *Phyllanthus emblica* (Pe) with and without coating of *Ocimum sanctum* (Os).

**Materials and methods:**

After obtaining Institutional Animal Ethics Committee approval, present experimental study was conducted to evaluate the immunomodulatory effects of plant drug formulations against infection induced in mice subjected to major surgical stress. Hemisplenectomy was selected to induce major stress and the procedure for hemisplenectomy was standardized. A model of secondary fungal infection after hemisplenectomy was established followed by the treatment of mice with plant drugs and controls. They were subjected to hemisplenectomy or sham operation and 10^5^ C. albicans were injected intravenously. The therapy continued for next 14 days. Kidneys were isolated to estimate fungal load. Fungal load of the kidneys was estimated on post-operative Day 15.

**Results:**

The test formulations Tc + Pe and Tc + Pe + Os showed significant reduction in the fungal burden of kidneys as compared to hemisplenectomized control group. However, Tc alone exerted better degree of protection as compared to Tc + Pe and Tc + Pe + Os.

**Conclusion:**

The formulations, Tc + Pe and Tc + Pe + Os that were developed on the basis of theoretical concepts were not found to be superior to Tc. Though the individual ingredients have been shown to possess immunnostimulant activities, in combination, Pe and Os blunted the effects of Tc. The basis for this drug interaction needs further exploration. Thus, the current experimental study validates immunomodulatory role of Tc. However, the addition of Pe with *bhavana* of Os does not lead to any augmentation of immunomodulatory activity of Tc. This study also underlines the need to generate data on Ayurveda formulations to understand the rationality of the multi-ingredient Ayurvedic formulations.

## 1 Introduction

The world has witnessed a pandemic which has changed life of every individual across the globe [1] leading to a shift in focus towards critical infectious diseases. Immunocompromised hosts whose immunologic mechanism is suboptimal constitute a significant proportion of the critically infected population [2]. Cases of fungal infections like mucormycosis post-COVID treatment has enhanced our focus on immunosuppression and its complications. In conditions like trauma, surgery, and radiation, stress-induced immunosuppression occurs. A human being encounters variety of stressors throughout the life. Surgical stress is one such important stressor. Ability to combat such stressors and maintain health involves interplay of the neuroendocrine system, immune system, and defense mechanisms of peripheral or target organs. In patients who undergo major surgery, immunosuppression has been reported as a leading cause of infection and is responsible for mortality and morbidity in the immediate post-operative period [[3], [4], [5]]. Our immune system is versatile and capable of recognizing and eliminating a variety of foreign invaders through a variety of defense mechanisms. A number of morphologically and functionally diverse organs and tissues participate in development of immune response. One of the important organs of immune system is spleen. The incidental reports mention a relationship between splenectomy and infection; it was not until 1952 that a causative association was reported between splenectomy (for congenital hemolytic anemia) and the occurrence of meningitis with sepsis. Since then, the increased risk of infection and septicemia directly related to splenectomy has been well-defined in the literature. Such infections are now generally termed overwhelming post-splenectomy infections (OPSI) [6].

In modern medicine, infections are treated using antimicrobial agents that inhibit or kill the microorganisms. With the advent of antimicrobial agents, it was believed that infectious diseases would soon be eliminated and become of historic interest only. However, the evolution of new infective diseases like COVID-19 and susceptibility of immunocompromised hosts to fatal opportunistic infections poses a greater challenge. Hence, it was felt necessary to search for agents that can be given to immunocompromised patients like patients with splenectomy to facilitate regeneration of spleen with preservation of its function and also have immunomodulatory activity. Ayurveda describes certain drugs which belong to *rasayana* group [7]. *Rasayana* drugs are known to have immunnostimulant properties. According to Ayurveda, these drugs also have rejuvenating potential as well. *Tinospora cordifolia* (Tc) a widely researched *rasayana* plant drug has shown to regenerate splenic cells in a model of hemisplenectomized mice and protect against *Candida albicans* [8]. However, many a times Tc is available in market in combinations with other drugs. One such formulation is that of Tc and *Phyllanthus emblica* (Pe). *P. emblica* is also a *rasayana* drug and as per Ayurveda, has affinity towards spleen [9]. *Ocimum sanctum* (Os) is yet another agent which has shown to enhance humoral antibody responses [10]. It is administered either as a fresh juice of leaves or is used to coat other plant drug particles and described as *bhavana*. As per Ayurveda and literature, *bhavana* increases the efficacy of the coated drugs [11,12].

Since the individual drugs Tc and Pe have shown to possess immunomodulatory effects, it was of interest to compare the immunomodulatory effects of combination of these with those of Tc alone. Hence, it was decided to evaluate the effects of the two formulations of Indian medicinal plants, Tc and Pe with or without Os (Tc + Pe and Tc + Pe + Os) on immunomodulatory function of spleen in hemisplenectomized mice and compared the same with that of Tc alone.

## 2 Material and methods

Permission from the Institutional Animal Ethics Committee from Seth G. S. Medical College and K. E. M. Hospital Mumbai (IAEC approval number: AEC/21/06) was taken before initiation of the study.

### 2.1 Animals

Total 48 male mice were selected for standardization of *C. albicans* dose and route of injection. Total 40 male mice were selected for the final study and divided into five treatment groups. Swiss albino mice of either sex weighing 25-35 g were used. These mice were procured from the in-house colony of the animal house. Study was conducted at Centre for animal studies and Ayurveda research centre at Seth G. S. Medical College and K. E. M. Hospital Parel, Mumbai, Maharashtra, India. Mice were maintained in accordance with the laboratory guidelines for the care and use of laboratory animals laid down by the Committee for the Purpose of Control and Supervision of Experiments on Animals (CPCSEA), India throughout the study period. The temperature and humidity in the experimental room were recorded once daily. Mice were housed in air conditioned rooms having 12–15 filtered fresh air changes per hour, 22 ± 3 °C temperature, and 30–70% relative humidity. Twelve hourly light and dark cycles were maintained. Mice were housed in a group of nine per cage in polypropylene cages. Autoclaved paddy husk served as the bedding. Cages were fitted with stainless steel top grill having facilities for providing food and water. They were given commercially available rodent food from Amrut Oil Mills Limited, Mumbai, and filtered and UV purified water *ad libitum*.

### 2.2 Drugs

Two plant drug formulations prepared using Tc, Pe and Os were tested. The first formulation contained Tc and Pe in a ratio of 1:3, while the second formulation had the same ingredients (Tc + Pe) but a coating with Os was given (*bhavana*). The doses of Tc and Pe were selected as advocated in Ayurveda and are expressed in terms of the crude powder of the dried stem/fruit, respectively [9]. These formulations were supplied by Shri Dhootapapeshwar Limited, Mumbai. The extractive values of both the formulations were 24.5%. These formulations were stored at room temperature in a desiccator. Whenever required, suspensions were made using 1% gum acacia as suspending agent.

Tc was selected as a positive control in this study. An aqueous extract of Tc was procured from Indian Institute of Integrative Medicine, Jammu.

All the study mediations were administered orally using a feeding tube in two divided doses given daily. The animals from vehicle control group were given equivalent volume of 1% gum acacia*.*

### 2.3 Hemisplenectomy

In this study, a model of hemisplenectomy - a major surgery, was selected to cause immunosuppression [13]. While selecting the animal model of major surgery, the literature revealed that the models of major surgery include amputation and gastrectomy. [14] However, mutilation and morbidity as a result of these surgeries is very high. [15] Such experiments are usually not permitted by the Animal Ethics Committee of the institute as they cause pain and permanent disability in animals. Cooney et al, in 1984 reported a model of major abdominal surgery in the form of hemisplenectomy, wherein, half of the spleen in the mice was removed. [16] Hence, for the present study it was decided to carry out hemisplenectomy in Swiss albino mice and find out whether they could survive for 30 days or more. A control was maintained as sham operated group (exteriorization of spleen for 1 min and then replacing it back into the peritoneal cavity) to rule out the effect of laparotomy and handling of the spleen on the stress parameters.

Thatte and Dahanukar have reported that strength of 10^6^ *C. albicans* injected intravenously to mice after 2 h of laparotomy resulted in 58% mortality after 10 days [17]. Hasenclever, in 1959 and Brieland et al.*,* in 2001 reported 100% mortality within seven days of intravenous injection of 10^7^ *C. albicans* in normal mice [18,19]. Literature search did not reveal any study wherein *C. albicans* was used to induce infection in hemisplenectomized mice.

Therefore, in the present study, four strengths of *C. albicans* (10^5^-10^8^) and two routes of administration (intravenous and intra-peritoneal) were selected to establish the optimum strength of *C. albicans* that would cause mortality in hemisplenectomized mice. It was decided to induce infection after 2 h of hemisplenectomy using the lowest strength intraperitoneally and find out the response of hemisplenectomized mice to induced infection [17]. No mortality was observed in any of the groups given 10^5^–10^8^ *C. albicans* intraperitoneally. These mice also did not show any signs of illness, i.e., loss of body weight, loss of appetite, loss of fur, etc. when observed for more than 30 days. Therefore, it was decided to inject *C. albicans* intravenously starting from the highest strength and decide the fungal strength to be injected based on the mortality within 10 days. Mice injected with 10^6^–10^8^ *C. albicans* died within 48 h. This early mortality in the hemisplenectomized mice indicated that they are more susceptible to *C. albicans* infection than normal and laparotomized mice and also points out to the underlying immunosuppression. The mice injected with the lowest strength (10^5^) intravenously survived beyond 10 days but exhibited signs of illness.

Firstly, a model of secondary fungal infection after major surgery was established using Swiss albino mice (6 animals/group/experiment). Six hemisplenectomized mice were randomly divided into 8 groups (n = 6/gp). These groups were injected with different strengths of *C. albicans* either intraperitoneally or intravenously. A step-wise approach was adopted as follows:

1. Selection and mastering technique of major surgery: Hemisplenectomy was selected as major surgery and done using technique described by Rao [8]. After confirming the survival of hemisplenectomized mice for 30 days, the next part was undertaken.
2. Selection of route and strength of secondary fungal infection: *C. albicans* was selected as the fungal agent. Four strengths (10^5^, 10^6^, 10^7^ and 10^8^ organisms/mouse) of *C. albicans* and two routes (intra-peritoneal and/or intravenous) were used. The infection was induced 2 h after hemisplenectomy. Survival data of these mice was reviewed. Fungal load of the kidneys of mice injected with 10^5^ *C. albicans* intravenously was determined on 14th day of infection by estimating colony forming units (CFU) from the isolated homogenized kidneys (diluted serially).
3. Confirmation of effects of Tc on CFU in the selected model: Two groups of mice (n = 6/gp) were administered either Tc (200 mg/kg/d orally) or vehicle for seven days. They were subjected to hemisplenectomy and 10^5^ *C. albicans* were injected intravenously. The therapy continued for next 14 days. Kidneys were isolated to estimate fungal load. Fungal load of the kidneys was expressed as CFU of *C. albicans* per ml of kidney homogenate.

**Table:** 

Groups (n = 6)	Concentrations of *C. albicans*	Route of injection
1	10^5^	Intraperitoneal
2	10^6^	
3	10^7^	
4	10^8^	
5	10^8^	Intravenous
6	10^7^	
7	10^6^	
8	10^5^	

### 2.4 Hemisplenectomy procedure

Mice were anaesthetized with thiopentone sodium 50 mg/kg intraperitoneally. After laparotomy, the spleen was identified and exteriorized. It was ligated midway and the caudal half of the spleen was excised. Blood loss was controlled by pressure homeostasis. After confirming adequate homeostasis, the remaining half of the spleen was replaced back into the abdomen. Peritoneal and the skin incision were sutured. Betadine dressing was applied. The mice were wrapped in gauze and kept in a polypropylene cage in warm environment. The animals were frequently monitored for any signs of bleeding from incision site and any signs of distress.

**Fig. 1:**
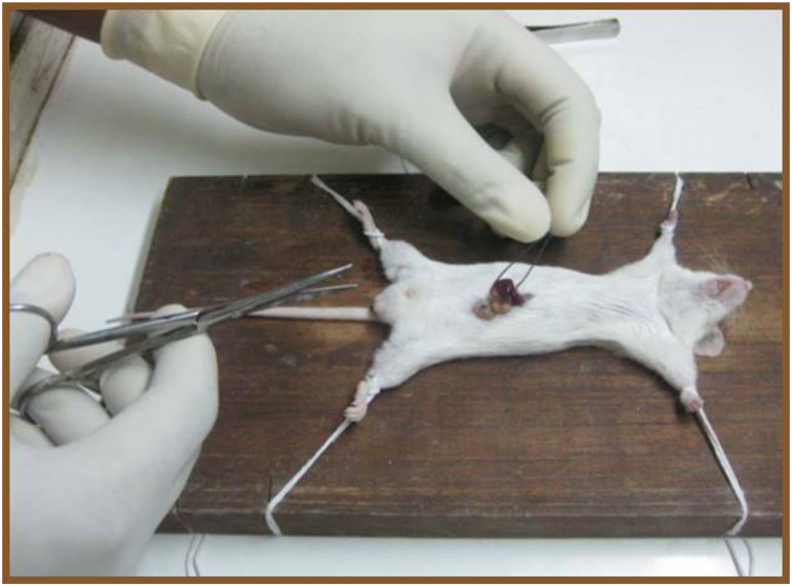
Hemisplenectomy.

A group of animals was operated same as hemisplenectomized animals except the spleen was exteriorized for 1 min and then replaced back into the abdomen without excision (Fig. 1). This group was designated as sham operated control. Total 48 mice were randomly assigned to 5 treatments and administered the drugs as given in Table 1.

**Table 1:** Experimental groups.

Group (n = 8)	Procedure	Vehicle/Drugs (mg/kg/day for 30 days)	Dose (mg/kg/day for 30 days)
1	Sham operated	Gum acacia (1%)	Equal volume (1 ml/kg/day)
2	Hemisplenectomy	Gum acacia (1%)	Equal volume (1 ml/kg/day)
3		Tc	100
4		Tc + Pe	400
5		Tc + Pe + Os	400

### 2.5 Methodology

Treatment with the drugs/vehicle was started from day 1 and given for 30 days. After 15 days of therapy, hemisplenectomy was carried out in Groups 2–6 whereas mice from group 1 underwent sham operation. Two hours later, 10^5^ *C. albicans* were injected intravenously. The respective therapy continued for the next 14 days (day 30). On day 30, kidneys were isolated and homogenized and processed further to estimate fungal load. Serial dilutions were prepared from the homogenate for plating on the agar plate. Fungal load was estimated by incubating the agar plates. Fungal load of kidneys was expressed as CFU of *C. albicans* per ml of kidney homogenate.

**Figure:**
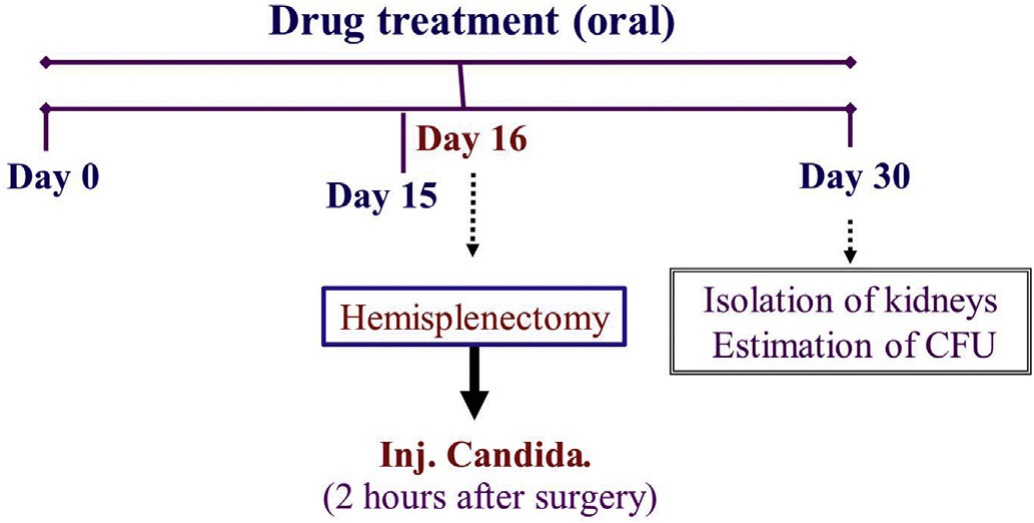

## 3 Results

In the initial phase, the model of major surgery viz. hemisplenectomy was standardized in mice. This was followed by standardization of the model of postoperative fungal infection in animals. Effects of the positive control, Tc were evaluated in this model. The results are presented below:

### 3.1 Selection and mastering technique of major surgery

Hemisplenectomy was selected as the model of major surgery. All the mice, in whom hemisplenectomy was carried out survived for more than 30 days as did the sham operated mice.

### 3.2 Selection of route and strength of secondary fungal infection

Intraperitoneal administration of 10^8^, 10^7^, 10^6^, 10^5^ *C. albicans*/mouse to the hemisplenectomized mice, after 2 h of surgery did not result in any mortality and all the mice survived for more than 30 days. On the other hand, those injected 10^8^, 10^7^, and 10^6^ *C. albicans* intravenously died within 48 h of injection. However, all the mice injected with intravenous 10^5^ *C. albicans* survived. From these mice, kidneys were isolated after 14 days to determine the fungal load. The colonies were countable (<300) only in the petri plates streaked with 1 in 10^7^, 1 in 10^8^, 1 in 10^9^ dilutions of kidney homogenates. The average computed CFU per ml of kidney homogenate for 6 mice were 1.52 ± 0.43 × 10^10^.

### 3.3 Confirmation of effects of Tc on CFU

The kidney homogenates obtained from hemisplenectomized animals showed more than 300 colonies when streaked undiluted and also in all the tested dilutions. In the Tc treated animals, more than 300 colonies were seen with undiluted homogenate and its 1 in 10 dilution. CFU per ml of kidney homogenate were calculated from other plates. The average CFU per ml of homogenate were 6 ± 0.53 × 10^9^. Thus, in surgically stressed mice, the fungal load in the kidneys of the mice treated with positive control was lower than that in the disease control animals.

A decision was taken to inject 10^5^ *C. albicans* by the intravenous route after 2 h of hemisplenectomy for induction of infection. In the surviving mice, on day 15 of infection, fungal load of the kidneys was estimated. The positive control Tc (200 mg/kg/day) was found to decrease the CFU of *C. albicans* per ml of kidney homogenates significantly. After the standardization, effects of the test formulations were evaluated against fungal infection induced in mice subjected to hemisplenectomy. The test formulations/vehicle were administered 15 days prior to surgery and continued for next 2 weeks.

**Fig. 2:**
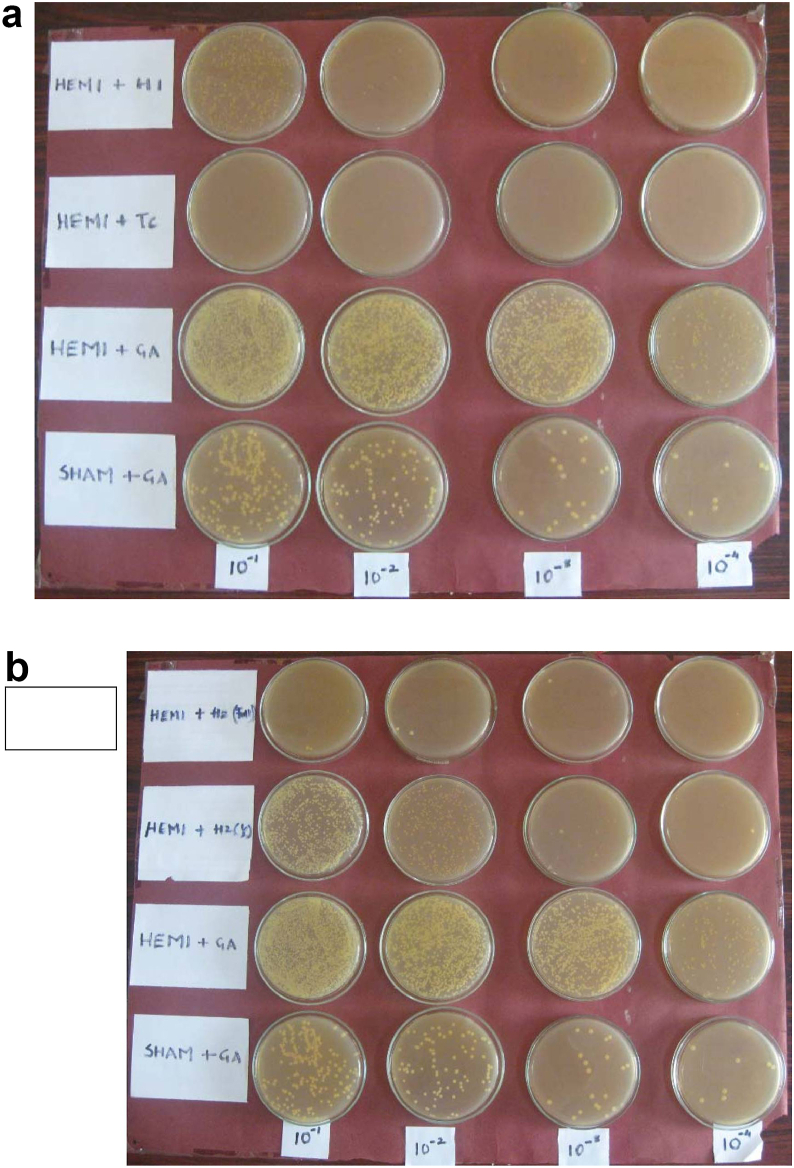
Fungal colonies from mice treated with different plant drugs.

**Fig. 3:**
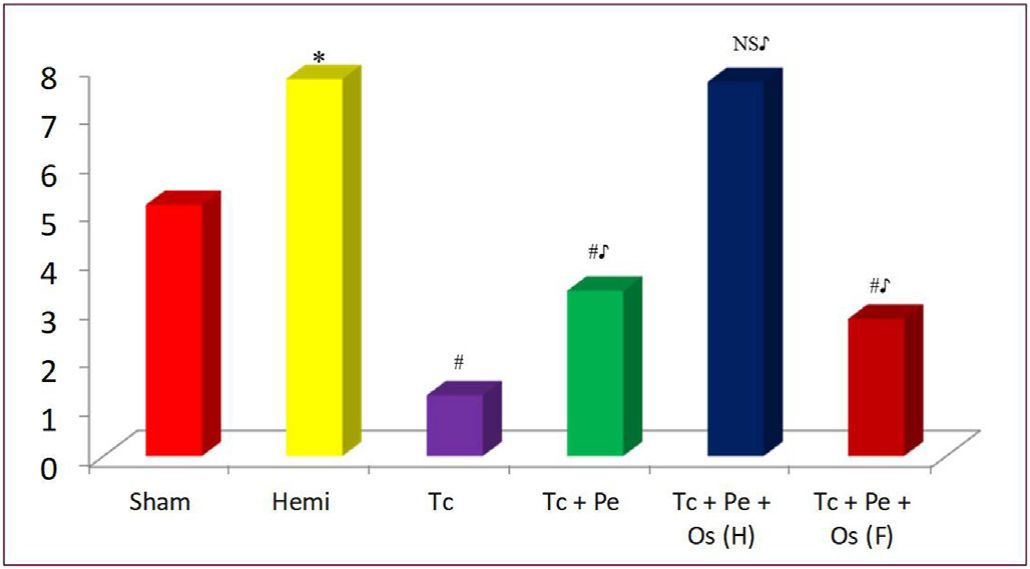
Comparison of kidney fungal loads in various groups. All figures represent Mean and SD; Unpaired *t* test * p < 0.001 vs Group 1 ANOVA followed post hoc Tukey test: # p < 0.001, NS = Not significant vs Group 2; ♪p < 0.001, NS2= Not significant vs Group 4.

Fungal loads after the treatments are shown in Fig. 2. In animals which underwent hemisplenectomy, the fungal load was significantly higher (p < 0.001) than that seen in the sham operated animals as seen in Table 2. Hemisplenectomized animals pre-treated with Tc showed a marked reduction in the fungal load and these values were significantly lower than the vehicle control. Both the test formulations reduced the fungal burden significantly as compared to the vehicle control (p < 0.001). However, their effects were significantly lesser than Tc treated group (p < 0.001). No reduction in the fungal load was observed after halving the dose of Tc + Pe + Os and the load in this group was comparable to the fungal load of the vehicle control group. Results are summarized in Fig. 3.

**Table 2:** Effect of various treatments (vehicle/study drugs) on the kidney fungal load in hemisplenectomized mice.

Sr. No.	Treatment Groups, Dose (mg/kg/d)	Procedure	Kidney fungal load (log_10_ CFU)
1	Vehicle	Sham operated	5.15 ± 0.2
2	Vehicle	Hemisplenectomy	7.74 ± 0.23∗
3	Tc*,* 100		1.24 ± 0.84∗^$^
4	Tc + Pe*,* 400		3.39 ± 0.26^$#^
5	Tc + Pe + Os*,* 400		2.82 ± 0.58^$#^

N = 8/group. All values shown are in Mean ± SD.

One way ANOVA followed by post hoc Tukey test ∗p<0.001 *vs* Group 1, ^$^p<0.001, NS= not significant *vs* Group 2, ^#^p<0.001 *vs* Group 3.

## 4 Discussion

The role of surgical stress in immune suppression is well-known [[2], [3], [4], [5]]. Clinical studies have shown that disseminated candidiasis is more common following major abdominal surgeries [20]. Severe infection itself causes immunosuppression [21,22]. It is clinically accepted that major surgery suppresses cell mediated immunity for several days and that more invasive procedures lead to deeper and longer immunosuppression. This immunosuppression can be quite profound, and many believe that it is a major factor in promoting life-threatening post-operative infections [15,21].

As mentioned in the results, survival at the end of 30 days was 100% for both hemisplenectomized and sham operated groups. This indicates that the mice recover from the operative stress without any mortality or morbidity and loss of half of the spleen does not cause any complications if the animals are maintained under the normal housing conditions of the animal house. The question remained whether there exist, an immunosuppression in hemisplenectomized animals. It has been reported that splenic immune functions are severely compromised only when more than 75% of the spleen is removed or damaged leading to increased susceptibility to various infections [13,23,24]. However, some researchers have also reported increased susceptibility to infection in hemisplenectomized mice as compared to normal mice demonstrating that the immune response is not optimal [23,25].

Hence, after mastering the technique of hemisplenectomy, it was decided to induce infection in hemisplenectomized mice. Clinical studies have shown that disseminated candidiasis is more common following major abdominal surgeries [20]. Elimination of *C. albicans* from an infected host requires the cooperation of many immune cells and their products. There is no evidence that antibody and complement can mediate the lysis of *C. albicans* ref, and phagocytes are probably the prime effector cells to prevent candidiasis [26]. As a part of reticuloendothelial system, spleen plays an important role in the defense against systemic candidiasis through its macrophages and ability to opsonize the fungi [26,27]. Hence *C. albicans* was selected as the insulting organism. The next concern was what should be the strength and the route administration of *C. albicans* organisms in the hemisplenectomized mice so that the resulting immunosuppression leads to at least 80% mortality.

Rajakaruna et al, showed that these plants do not have anti-fungal effects. [28] Hence, one option was to study the effects of the test formulations in combination with sub-therapeutic doses of anti-fungal agents against the lethal concentrations of *C. albicans* and find out whether they potentiate the anti-fungal effects by boosting immune system. This option was discarded as the literature search revealed that anti-fungal agents themselves have an immunomodulatory effect, which could act as a confounding factor while evaluating the test formulations. [29,30]

Studies have shown that after systemic candidial infection, the fungi localize to the kidneys. [17,31] Lionakis et al reported that in a mouse model of invasive candidiasis, fungal burden changes with variable dynamics in the kidney, brain, spleen, and liver and declines in all organs except for the kidneys. [32] It has also been reported that 10^5^ *C. albicans* injected intravenously required a period of around two weeks for colonization in kidneys. [29,33] Hence, it was felt that in the present study, in the surviving mice given 10^5^ fungal load intravenously, fungal load in the kidneys should be estimated on day 15 of surgery. If the fungal burden was high, then it could serve as a surrogate marker for immunosuppression secondary to surgical stress. When serial dilutions of kidney homogenates were studied, all dilutions showed presence of fungal colonies. This proved that hemisplenectomized mice injected with 10^5^ *C. albicans* intravenously had high fungal load in the kidneys.

As a next step it was decided to confirm the effects of the positive control Tc, against the fungal load in these mice. Although Tc has been shown to reduce the mortality after systemic candidial infections, its effects on the fungal load has not been reported [22]. To shorten the period of standardization, therapy with 200 mg/kg of Tc was given for 7 days prior to surgery. This dose and duration produces effects similar to those with 100 mg/kg given for 14 days [34,35]. In the control group, as observed earlier there was a massive growth of colonies in almost all dilutions; on the other hand, kidney homogenates of Tc treated mice did not show any growth beyond 1 in 10^3^ dilutions. These results confirmed that the model was standardized. Further it was decided to use only five serial dilutions of kidney homogenates ranging from 1 in 10 to 1 in 10^5^ to detect the effects of test drugs. Having established two different models of stress-induced immunosuppression, experiments were undertaken to evaluate effects of the study drugs. The selected test drug formulations were administered orally to the animals before exposing them to stress as per the principles of pro-host therapy [36]. As there is no pharmacokinetic data available for plant drugs, it was decided to pre-treat the animals with the drugs to ensure attainment of adequate concentrations at the site before subjecting them to the insult. The duration of pre-treatment was selected as 15 days prior to the experimental procedure as Tc given in a dose of 100 mg/kg exhibits its effects by 15 days [7]. The treatment was continued for another 14 days to maintain the concentrations in the post-operative period.

The test formulations showed significant reduction in the fungal burden of kidneys as compared to hemisplenectomized control group. However, Tc alone exerted better degree of protection. Thus, the formulations Tc + Pe and Tc + Pe + Os that were developed on the basis of theoretical concepts were not found to be superior to Tc. The immune stimulatory activity is also reflected in protection against the *C. albicans* infection. Both the formulations reduced the kidney fungal load. However, Tc alone was found to offer better degree of protection. This may be due to macrophage stimulatory activity of Tc rather than effect on B lymphocytes.

Earlier Alrumaihi and colleagues have reported that extracts of Tc has the potential to reinvigorate the debilitated immune system and eliminate systemic candidiasis in mice [37]. Results from the present study affirms this observation. Multiple studies have earlier highlighted the immunomodulatory role of Tc [[7], [8], [9],^34^,^35^,[37], [38], [39], [40], [41], [42]].

The multi-ingredient formulations stated in Ayurveda (using the concept of *pathsanyojana*) have evolved through the intellectual exercise as well as the clinical experience of physicians [9]. Concept of combining the *rasayana* is also already established and well-studied [9,11,12]. If combinations of Ayurvedic drugs are to be developed today, they should be backed by the rigorous scientific studies not only to generate evidence regarding their efficacy and safety but also their superiority over the single plant drugs incorporated in them. The present study is an example of an approach that can be adopted while developing new formulations of drugs from Ayurveda. It also highlights the importance of comparing the combinations with the single plant drugs incorporated in them. Such an approach will prevent the flooding of the market with proprietary multi-ingredient Ayurvedic formulations which may be effective and safe but not superior to individual plant drugs and offer no added advantage. The government of India and Ministry of AYUSH is taking progressive strides towards enhancement and growth of Ayurveda with rational use of Ayurveda formulations. [43,44] Research and development towards the validation of Ayurveda is being projected as the thrust area. Hence, such studies are warranted.

Thus, the present study substantiates the immunomodulatory role of Tc; however, addition of Pe with *bhavana* of Os didn't result in any enhancement of immunomodulatory activity of Tc. Further studies are needed to explore the immunomodulatory role of combination of *rasayana* drugs. It will also be worthwhile to see its effects in clinical trials.

## 5 Conclusion

In the present study the formulations, Tc+Pe and Tc+Pe+Os, were not found to be superior to Tc in terms of immunomodulatory activity. This highlights the point that theoretical concepts mentioned in Ayurveda need to be validated with proof of concept studies. The present study also underlines the need to have a strict regulation of plant drugs whereby polyherbal formulations are marketed only after comparing their effects against the single plant drug incorporated therein. Current situation demands rational use of traditional medicines.

## Source(s) of funding

The grant for this study was received from Research Society, Seth G. S. Medical College and K. E. M. Hospital, Parel Mumbai - 400 012, Maharashtra, India.

## Conflict of interest

None.

## Author contributions

DM searched literature and conceived the study. DM was involved in protocol development, gaining ethical approval, study procedures and data analysis. HM and DM wrote the first draft of the manuscript. AM, HM gave inputs on results and expert opinion on discussion. All the authors provided valuable suggestions on answering the queries from journal.
